# Two-photon microscopy for microrobotics: Visualization of micro-agents below fixed tissue

**DOI:** 10.1371/journal.pone.0289725

**Published:** 2023-08-10

**Authors:** Juan J. Huaroto, Luigi Capuano, Mert Kaya, Ihar Hlukhau, Franck Assayag, Sumit Mohanty, Gert-willem Römer, Sarthak Misra

**Affiliations:** 1 Surgical Robotics Laboratory, Department of Biomechanical Engineering, University of Twente, Enschede, The Netherlands; 2 Surgical Robotics Laboratory, Department of Biomedical Engineering, University Medical Centre Groningen and University of Groningen, Groningen, The Netherlands; 3 Animal Facility, Technical Medical Centre (TechMed Centre) Infrastructure, Faculty of Science and Technology, University of Twente, Enschede, The Netherlands; 4 Autonomous Matter Department, AMOLF, Amsterdam, The Netherlands; 5 Chair of Laser Processing, Department of Mechanics of Solids, Surfaces & Systems (MS3), Faculty of Engineering Technology, University of Twente, Enschede, The Netherlands; Istituto Italiano di Tecnologia, ITALY

## Abstract

Optical microscopy is frequently used to visualize microrobotic agents (i.e., micro-agents) and physical surroundings with a relatively high spatio-temporal resolution. However, the limited penetration depth of optical microscopy techniques used in microrobotics (in the order of 100 μm) reduces the capability of visualizing micro-agents below biological tissue. Two-photon microscopy is a technique that exploits the principle of two-photon absorption, permitting live tissue imaging with sub-micron resolution and optical penetration depths (over 500 μm). The two-photon absorption principle has been widely applied to fabricate sub-millimeter scale components via direct laser writing (DLW). Yet, its use as an imaging tool for microrobotics remains unexplored in the state-of-the-art. This study introduces and reports on two-photon microscopy as an alternative technique for visualizing micro-agents below biological tissue. In order to validate two-photon image acquisition for microrobotics, two-type micro-agents are fabricated and employed: (1) electrospun fibers stained with an exogenous fluorophore and (2) bio-inspired structure printed with autofluorescent resin via DLW. The experiments are devised and conducted to obtain three-dimensional reconstructions of both micro-agents, perform a qualitative study of laser-tissue interaction, and visualize micro-agents along with tissue using second-harmonic generation. We experimentally demonstrate two-photon microscopy of micro-agents below formalin-fixed tissue with a maximum penetration depth of 800 μm and continuous imaging of magnetic electrospun fibers with one frame per second acquisition rate (in a field of view of 135 × 135 μm^2^). Our results show that two-photon microscopy can be an alternative imaging technique for microrobotics by enabling visualization of micro-agents under *in vitro* and *ex ovo* conditions. Furthermore, bridging the gap between two-photon microscopy and the microrobotics field has the potential to facilitate *in vivo* visualization of micro-agents.

## Introduction

The field of microrobotics has the potential to revolutionize the traditional way of performing surgery by steering microrobotic agents within confined anatomical structures [1, 2]. Microrobotic agents (i.e., micro-agents) are the means to achieve tasks such as drug delivery, biopsy, and micro-assembly of biological samples [3, 4]. Typically, these miniaturized agents are responsive to external stimuli (e.g., magnetic fields [5], acoustic waves [6], and light [7–9]) and necessitate sensing/detection strategies for navigation and feedback. The development of novel techniques for visualization of micro-agents holds importance to translate the microrobotics field to the clinics and expand the application domains to clinically-relevant scenarios [4, 10, 11]. Visualization or imaging is required in microrobotics because the traditional sensors cannot be attached to the micro-agents due to size limitations. Thus, imaging modalities are used as external sensors to validate the functionalities and provide feedback. The literature on imaging techniques for microrobotics identifies three essential components towards visualization of micro-agents under *in vivo* and *in vitro* conditions. (1) Visualize micro-agents and physical surroundings [12]. (2) Achieve relatively high spatio-temporal resolution and penetration depth [13]. (3) Reduce the risk of side effects on patients/clinicians [14].

Current imaging techniques for the application domains of microrobotics partially undertake all three essential components. Ionizing techniques (e.g., X-ray computerized tomography [15], positron emission tomography [16], fluoroscopy [17], and single-photon emission computerized tomography [18]) have been proved for visualization of micro-agents towards *in vivo* applications. Nevertheless, the risk of side effects on patients and clinicians encourages the development of non-ionizing imaging techniques for microrobotics. Optical imaging [12, 19, 20], ultrasound [21], magnetic resonance imaging [22], photoacoustic imaging [11, 23], and magnetic particle imaging [24] are non-ionizing techniques used for visualization of micro-agents and physical surroundings. Optical imaging techniques such as bright field, confocal, and fluorescence microscopy offer a relatively high spatio-temporal resolution compared to other non-ionizing imaging techniques [14]. Yet, the limited optical penetration depth, scattering, and background noise reduce the capability of visualization micro-agents below biological tissue [19, 25]. Among non-ionizing techniques, photoacoustic imaging (i.e., microscopy and tomography) is a hybrid technique applicable to the field of microrobotics, addressing the limited penetration depth commonly encountered in optical microscopy techniques. For example, photoacoustic microscopy enables imaging of micro-agents underneath shallow tissue regions (<~ 1 mm) with a relatively high spatial resolution (3.2 μm) [8]. Photoacoustic tomography can overcome the penetration depth of photoacoustic microscopy. However, there exists a trade-off between spatial resolution and penetration depth. The spatial resolution achieved by photoacoustic tomography is typically in the order of 100 μm, which can hinder the detailed visualization of micro-agent morphology and intricacies [10, 11, 14]. Furthermore, the distortion of wavefronts caused by the non-uniform speed of sound in biological tissues can significantly impact the imaging performance under *in vivo* conditions [26].

An appealing remedy to overcome the shortcomings of optical microscopy techniques in microrobotics is two-photon microscopy [14, 27]. This non-ionizing fluorescence technique uses a femtosecond pulsed laser to generate two-photon absorption, allowing for visualization of biological tissue with penetration depths over 500 μm [28, 29]. The phenomenon of two-photon absorption can also trigger autofluorescence and second-harmonic generation in biological tissue samples, permitting stain-free imaging [30]. The spatial and temporal resolutions achieved through two-photon microscopy are in the order of sub-microns and 26.4 frames per second (in the field of view of 1490 × 128 pixels), respectively [13]. Although two-photon absorption has found applications for fabricating sub-millimeter size components via direct laser writing (DLW) (e.g., micro-agents [31], miniaturized optical lenses [32], and metamaterials [33]), its use in microrobotics for visualizing miniaturized agents below biological tissue remains unexplored in the literature.

This study aims to introduce two-photon microscopy as an alternative imaging tool to visualize micro-agents below biological tissue. Two types of fluorescent micro-agents are fabricated and used for visualization. (1) A bio-inspired structure (called CeFlowBot) printed with an autofluorescent resin (IP-Dip resin) via DLW [31]. (2) Electrospun fibers (i.e., beaded fibers and rod-like particles) stained with an exogenous fluorophore (coumarin 6) [12]. The biological tissues used throughout the study are prepared from rat organs and White Leghorn chick embryos according to protocols for bio-products and the Dutch animal care guidelines. The rat organs are fixed to preserve cells and tissue morphology long-term. The remainder of this study presents and discusses five experimental protocols. (1) The spectrum analysis of the fluorescent micro-agents. (2) Two-/Three-dimensional visualization of micro-agents. (3) Qualitative study of laser-tissue interaction. (4) Visualization of micro-agents below fixed tissue. (5) Continuous visualization and magnetic actuation of electrospun fibers.

## Materials and methods

### Experimental validation platform

**Fig 1:**
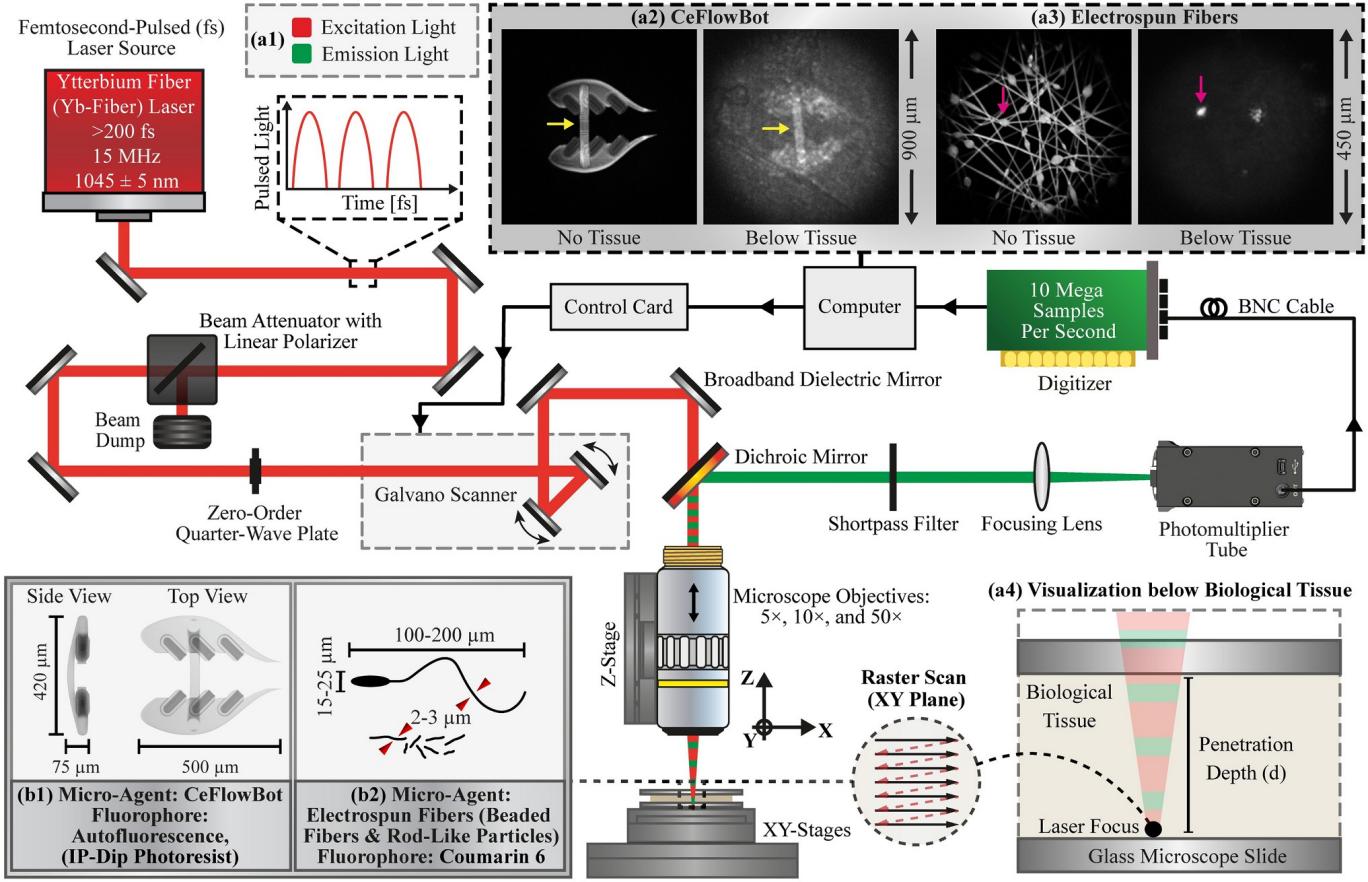
Schematic showing the optical layout of the two-photon microscope developed for visualization of micro-agents along with biological tissue. (a1) The microscope utilizes a femtosecond laser for excitation light generation while the emission light is delivered to a photomultiplier. The following micro-agents are visualized below biological tissue: (a2) a bio-inspired structure called CeFlowBot, and (a3) electrospun fibers. The colored arrows indicate structural morphologies of the micro-agents that are visible below biological tissue. (a4) Illustration of the samples used to visualize micro-agents below biological tissue. (b1)-(b2) Schematic representation of the fabricated fluorescent micro-agents.

We commence this study by describing the two-photon microscope developed to visualize micro-agents along with biological tissue (Fig 1). This microscope uses a Ytterbium fiber (Yb-fiber) femtosecond pulsed laser (Y-Fi, KMLabs, USA) as an excitation light source to generate fluorescence emission light from the samples (Fig 1(a1)). The laser beam is characterized by ultra-short pulses (> 200 fs) with a repetition rate of 15 MHz and a center wavelength (*λ*) of 1045 nm. The energy of the laser pulses is modulated using a beam power attenuator (Ultrafast, Altechna, Lithuania), which also generates a linear polarized beam. Next, a zero-order quarter-wave (WPQSM05–1030, Thorlabs, USA) creates a circularly polarized beam for image contrast enhancement [34]. Three infinity-corrected microscope objectives (M Plan Apo, Mitutoyo, Japan) with different magnifications (5×, 10×, and 50× magnification) are used to focus the beam and collect the emission light from three fields of view (900 × 900 μm^2^, 450 × 450 μm^2^, and 90 × 90 μm^2^, respectively). An XY-stage (ALS130–150, Aerotech, USA) with a positioning resolution of 100 nm holds and moves the sample on the plane. A linear stage (ATS100, Aerotech, USA) with a positioning resolution of 500 nm is employed to manipulate the microscope objective along the Z-axis and adjust the focal plane. In order to perform a raster scan, a control card (RTC4, SCANLAB GmbH, Germany) is used to rotate the mirrors of a Galvano scanner (IntelliScan14, SCANLAB GmbH, Germany) and steer the laser beam. Next, the laser beam is oriented through the microscope objective and focused on the sample for excitation. Fig 1(a4) shows the samples used in the experiments, in which micro-agents are placed below biological tissue. The excitation light (laser beam) delivered by the microscope objective is dispersed due to the optical components, stretching the pulse length. The pulse length is measured (~ 230 fs) after the excitation light passes through the microscope objective using an autocorrelator (pulseCheck, APE Angewandte Physik und Elektronik GmbH, Germany). The emission light generated by the sample passes back through the microscope objective and is reflected by the dichroic mirror (DMLP805, Thorlabs, USA) in the wavelength range of 400–785 nm. The reflected emission light passes through a short pass filter (FESH0750, Thorlabs, USA) (transmission range of 400–740 nm) and a focusing lens (LA1509-A, Thorlabs, USA) before being collected with a photomultiplier tube (PMT1001/m, Thorlabs, USA) for image formation (Fig 1(a2) and 1(a3)). For magnetic actuation of magnetic electrospun fibers, a needle-shaped electromagnetic coil is fabricated and employed [35]. The electromagnetic coil can generate 9 mT and 3.5 T/m at 5 mm from the tip axis by powering the coil at 1 A with a power source (E36313A, Keysight, USA).

### Fluorescence data acquisition

The fluorescence data is acquired synchronously while steering the laser beam (50 mm/s scanning speed) with the Galvano scanner (IntelliScan14, SCANLAB GmbH, Germany). The voltage signal produced by the photomultiplier tube (PMT1001/m, Thorlabs, USA) in response to the varying fluorescent light is digitized using a data acquisition card (ATS 9146, Alazartech, Canada) that allows for the acquisition and transmission of data simultaneously (10 MS/s sampling rate). The data is acquired in dark room conditions to avoid direct light contamination. The data obtained in each raster scan cycle (Fig 1(a4)) is converted into a gray-scale image for analysis. Since the two-photon microscope has a single photomultiplier channel, 16-bit gray images (over 65000 shades) are used for analyzing micro-agents along with the tissue.

### Image analysis

The gray-scale images (raw images) acquired from the two-photon microscope are analyzed offline through a MATLAB (version R2022b, MathWorks, USA) custom script. Since the two-photon microscope has a single photomultiplier channel, the analysis of micro-agents along with biological tissue relies on the difference in fluorescence intensities. We implement a contrast analysis based on contour, gradient, and intensity maps normalized to the corresponding maximum values. Although the microscope has a single photomultiplier channel, the three maps provide information about the micro-agents and surroundings. For continuous visualization of magnetic electrospun fibers, we acquired and stored the images using an acquisition rate of 1 frame per second in a field of view of 135 × 135 μm^2^. Thereon, the motion analysis is carried out offline using the Lucas-Kanade optical flow method [36]. The results of motion analysis are expressed as vector fields, which provide information on the motion of magnetic electrospun fibers [12, 37].

### Fabrication of micro-agents

The CeFlowBot is fabricated from an autofluorescent photoresist (IP-Dip, Nanoscribe GmbH, Germany) via dip-in laser lithography (DiLL) using a 25× microscope objective (0.8 numerical aperture) (Fig 1(b1)) [31]. The CeFlowBot samples are micro-printed on a glass substrate coated with Indium-tin oxide using a laser lithography system (Photonic Professional GT2, Nanoscribe GmbH, Germany) in Galvano scanning mode with a laser power of 25 mW, a scanning speed of 4 × 10^4^ μm/s, and slicing/hatching distances equal to 1 μm. The 3D printed structure is developed in cleaning solvent (RER 600) for 25 min and rinsed in isopropyl alcohol for 5 min. Thereon, the substrate containing CeFlowBot samples is baked in a hot plate (150°C) for 15 min to remove the photoresist residues.

Electrospun fibers (i.e., beaded fibers and rod-like particles) are fabricated using the electrospinning technique (Fig 1(b2)) [12]. The polymer solution for electrospinning consists of polystyrene pellets (430102–1KG, Sigma-Aldrich, USA) as a carrier polymer, coumarin 6 (442631–1G, Sigma-Aldrich, USA) as a hydrophobic fluorophore, iron oxide (Fe_3_O_4_) nanoparticles (637106–25G, Sigma-Aldrich, USA) as a magnetic material, and anhydrous N, N-Dimethylformamide (DMF) as a solvent (227056–1L, Sigma-Aldrich, USA). For beaded fibers (fluorescent micro-agents), a polymer solution is prepared using 30.0% (29.8% polystyrene and 0.2% coumarin 6) weight-to-volume ratio in DMF. For rod-like particles (fluorescent and magnetic micro-agents), the polymer solution is prepared using 45.0% (29.8% polystyrene, 15% Fe_3_O_4_, and 0.2% coumarin 6) weight-to-volume ratio in DMF. Each polymer solution is homogenized by stirring for 12 hours using a vertical roller (LLG-uniRoller 6, LLG-Labware, Germany). Each solution (0.04 mL) is electrospun at once using an accelerating voltage of 14 kV, a feed rate of 2.5 mL/h, and a needle tip-to-collector distance of 16 cm. Randomly oriented fiber meshes are collected on a grounded aluminum foil at 24°C and 22% humidity and dried at room temperature for 12 hours to remove the solvent residue. Fluorescent and magnetic electrospun fibers are obtained by grinding the fibers using a sonicator (model 2510, Branson, USA) for 2 hours. Magnetic electrospun fibers are prepared for continuous image acquisition using 1% (volume/volume) Tween 80 (P4780–100ML, Sigma-Aldrich, USA) in Milli-Q water.

### Scanning electron microscopy of micro-agents

A scanning electron microscope (JSM-7200F, JEOL, Japan) is utilized to obtain micrographs, which provide visual information about the morphology of the micro-agents. The microscopy parameters for imaging micro-agents (i.e., beaded fiber and CeFlowBot) include magnification, working distance, and acceleration voltage. The micrograph of beaded fiber is acquired using 6000× magnification, 9.2 mm working distance, and 5 kV acceleration voltage (Fig 3(a3)). For the micrograph of CeFlowBot, we use 150× magnification, 4 mm working distance, and 15 kV acceleration voltage (Fig 3(b3)).

### Spectrum analysis of fluorophores

The spectrum analysis of the fluorophores utilized in the micro-agents is performed using a spectrofluorometer (FP-8300, Jasco, Japan). The exogenous fluorophore is prepared for analysis using a solution of 4 μg of coumarin 6 (442631–1G, Sigma-Aldrich, USA) in 700 μL dimethylformamide (227056–1L, Sigma-Aldrich, USA) [12]. The autofluorescent resin is prepared for analysis using a solution of 0.14 μL of IP-Dip resin (IP-Dip, Nanoscribe GmbH, Germany) and 700 μL of Isopropyl alcohol (20842.312, VWR International, USA). Both solutions are transferred into separate quartz cuvettes (CV10Q700F, Thorlabs, USA) for spectrofluorometry using wavelength intervals of 1 nm.

### Tissue preparation

The rat tissues used in the experiments are taken post-mortem from outbred albino naïve female rats (RjHan: WI, Janvier Labs, France). Organs and biological samples are prepared and disposed of according to the Cat.1 directive for animal bio-products. The rat organs are fixed using a 10% buffered formalin solution (Formalin 10% Qpath Ref: 11699404 from VWR) overnight. Thereon, the organs are immersed in 100% ethanol for 24h and finally stored dry at 4°C. The rat tissues are accurately sliced with a scalpel and placed between two glass microscope slides for flattening the surface. For static image acquisition experiments, the tissue thickness is measured by introducing standard spacers of known thickness between the glass microscope slides (Fig 1(a4)). For continuous visualization below the rat ileal wall, the tissue thickness is measured by placing the spacers between a glass microscope slide and the microfluidic channel.

The *ex ovo* chorioallantoic membranes are obtained from incubating White Leghorn chick embryos at 38°C and 65% humidity throughout the culturing process [12]. At the embryonic day of development (EDD) 10, the egg contents are cracked into 60 mm cell culture Petri dishes for imaging. According to the Dutch animal care guidelines, Institutional Animal Care and Use Committee (IACUC) approval for chicken embryo experimentation is not necessary unless hatching is expected. Moreover, only experiments with chick embryos of development EDD14 and older need IACUC approval. The embryos used in this study are all in the early stages of embryo development (EDD10). Fertilized chicken eggs used in this study are purchased from approved poultry egg farms in the Netherlands.

### Tissue damage by laser ablation

The laser beam is focused on the fixed tissue (rat liver dissection). Thereon, a Galvano scanner (IntelliScan 14, SCANLAB GmbH, Germany) steers the laser beam along a straight line of 400 μm length with a speed of 50 mm/s, repeating this process five hundred times. The laser-affected tissue is imaged using a confocal microscope (Zeiss LSM 880, Carl Zeiss AG, Germany) with a 20× microscope objective (LD Plan-Neofluar, Carl Zeiss AG, Germany). The average power of the femtosecond pulsed laser is characterized by the laser power percentage (LPP), which is modulated by a beam attenuator (Ultrafast, Altechna, Lithuania). A photodiode power sensor (S130VC, Thorlabs, USA) measures the average laser power at the focal plane of a 10× microscope objective (M Plan Apo, Mitutoyo, Japan).

## Results

### Spectrum analysis of fluorescent micro-agents

**Fig 2:**
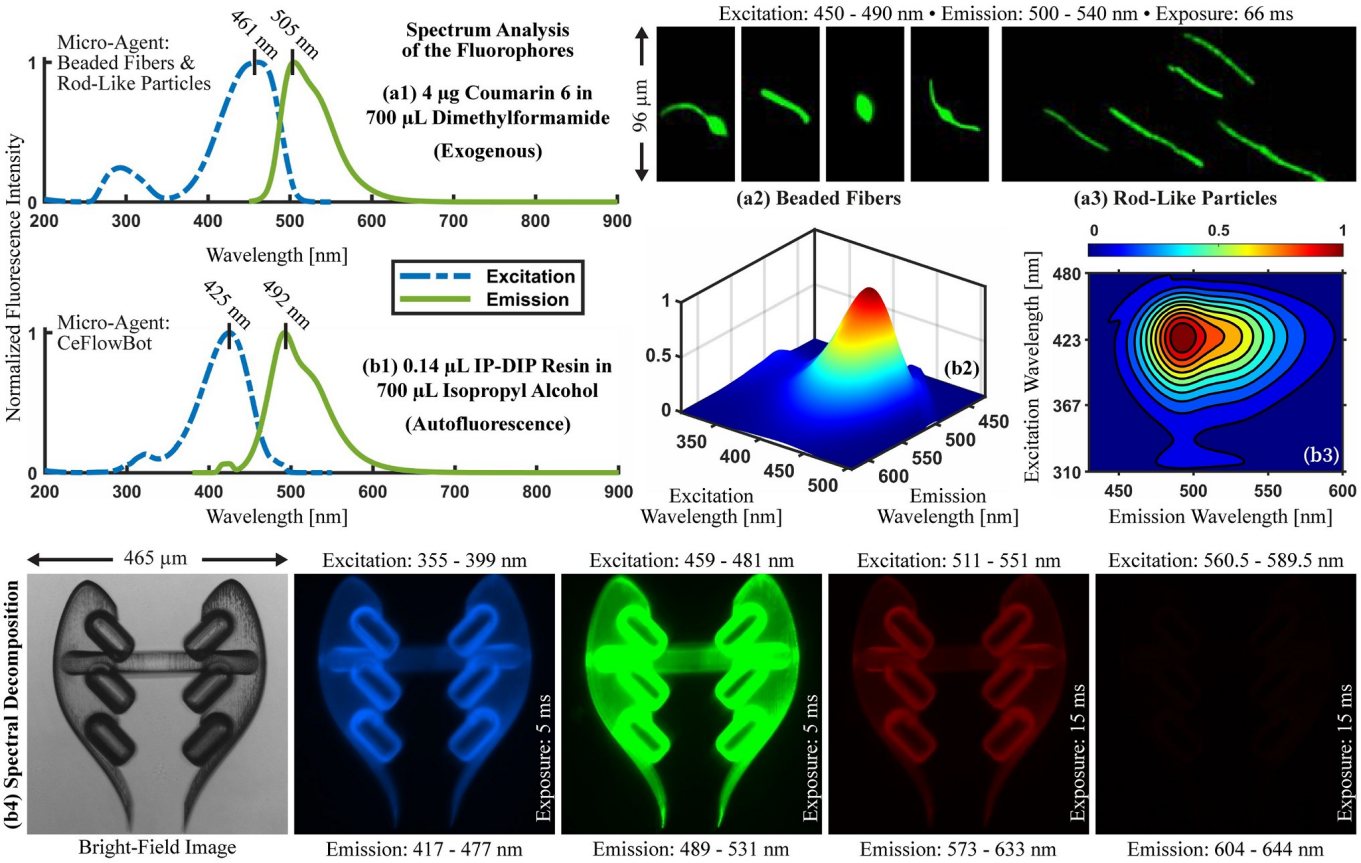
Spectrum analysis of fluorophores. (a1) Coumarin 6 (exogenous) (442631–1G, Sigma-Aldrich, USA) and (b1) IP-Dip resin (autofluorescence) (IP-Dip, Nanoscribe GmbH, Germany). Fluorescence microscopy of electrospun fibers with the following types of morphology: (a2) beaded fibers and (a3) rod-like particles. (b2)-(b3) 3D and contour plots showing the spectrum of IP-Dip resin at varying excitation and emission wavelengths. (b4) Spectral decomposition of CeFlowBot (made from IP-Dip resin) using a fluorescence microscope (EVOS FL, Life Technologies, USA).

The first step in two-photon microscopy of fluorescent micro-agents is the spectrum analysis of fluorophores used for staining or fabricating micro-agents. The spectrum analysis of fluorophores provides the excitation/emission wavelength limits for fluorescence imaging. Fig 2(a1) shows the spectrum analysis of coumarin 6 (exogenous) used to stain electrospun fibers (i.e., beaded fibers and rod-like particles). Coumarin 6 exhibits normalized fluorescence intensity peaks (excitation/emission) of 461 nm and 505 nm, respectively. For validating the spectrum analysis of coumarin 6, electrospun fibers are imaged using a custom-built fluorescence microscope [25] with excitation light wavelengths (450–490 nm) (Fig 2(a2) and 2(a3)).

The second fluorophore used in this study is a photoresist (IP-Dip) in the form of an autofluorescent resin widely used for fabricating sub-millimeter components for microrobotics via DLW [38]. Previous studies on excitation/emission spectra of IP-Dip resin provide the emission spectrum for a limited range of wavelengths [39, 40]. Here, we present the ultraviolet and visible spectra of IP-Dip resin at varying excitation and emission wavelengths (Fig 2(b1)–2(b3)). The IP-Dip resin show normalized fluorescence intensity peaks (excitation/emission) of 425 nm and 492 nm, respectively. In order to validate the spectrum analysis of IP-Dip, we perform the spectral decomposition of CeFlowBot using a commercial fluorescence microscope (EVOS FL, Life Technologies, USA). The spectral decomposition permits the identification of the excitation light wavelengths for fluorescence imaging. Fig 2(b4) shows that excitation light in the wavelength range (355–551 nm) can generate fluorescence image acquisition with exposure duration of 5 ms and 15 ms.

### Two-photon microscopy of micro-agents

The spectrum analysis and spectral decomposition of fluorescent micro-agents determine the wavelength limits of excitation/emission light for fluorescence image acquisition. However, it is often difficult to predict the two-photon excitation spectra from one-photon data because of the different quantum mechanical backgrounds [41]. In order to experimentally validate two-photon microscopy of fluorescent micro-agents characterized by one-photon excitation/emission spectra, we set the photomultiplier at maximum gain. The laser power percentage (LPP) is gradually increased until obtaining image formation. Our results show that a minimum of 10% LPP (6 mW average power at 15 MHz repetition rate) is required for visualization of the structural morphologies of micro-agents. Fig 1(a2) and 1(b3) show two-photon image acquisition of both micro-agents using 20% LPP (35 mW average power).

**Fig 3:**
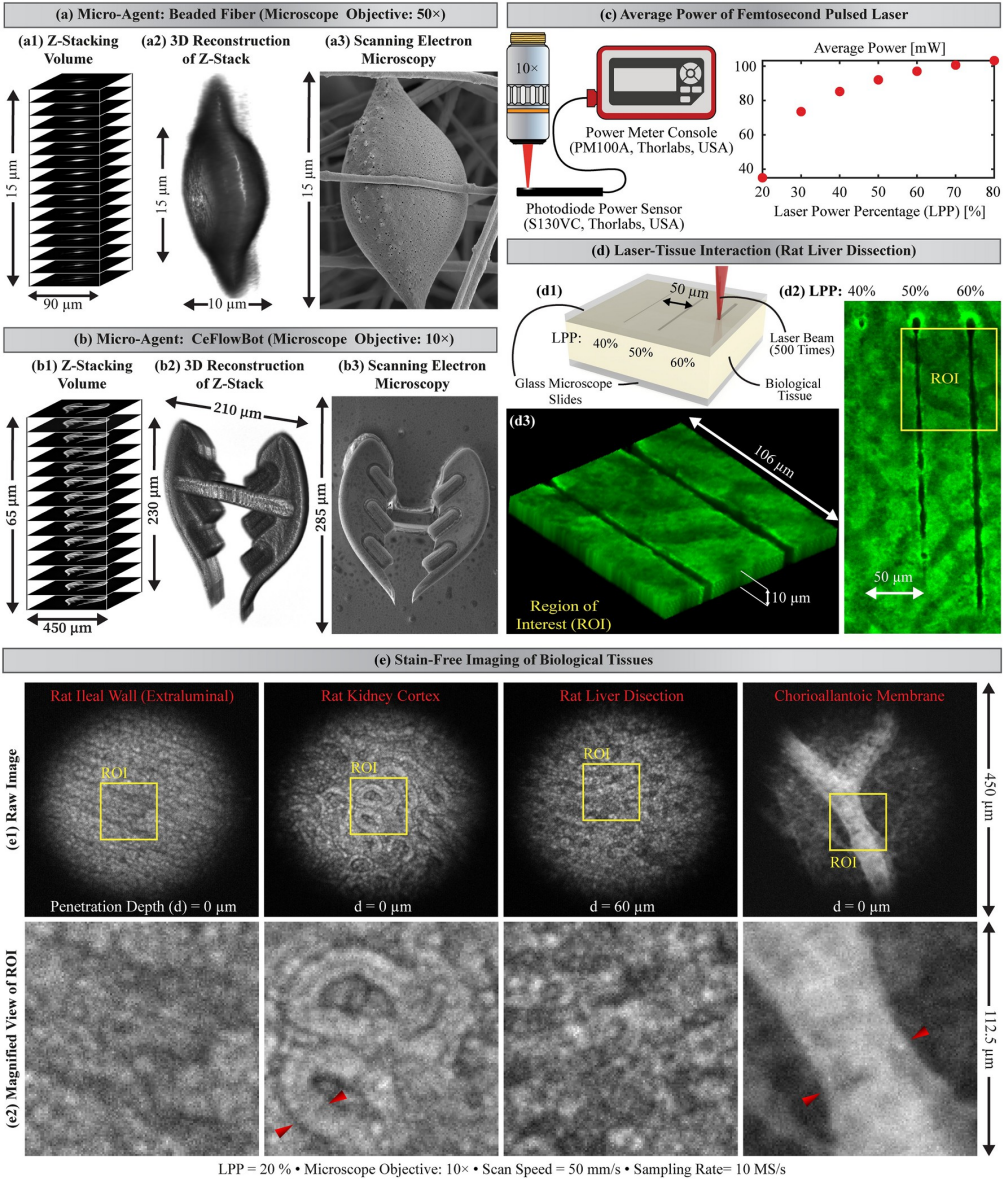
Z-stacking, 3D reconstruction, and scanning electron microscopy of micro-agents. (a1)-(a3) beaded fiber and (b1)-(b3) a scaled version of the bio-inspired structure (CeFlowBot), respectively. (c) Average power of femtosecond pulsed laser (pulse width of ~ 230 fs and repetition rate of 15 MHz) at the focus of the laser beam. (d1) Illustration of the laser-tissue interaction using three laser power percentages (LPPs): 40%, 50%, and 60% with the corresponding average powers of 85 mW, 92 mW, and 97 mW, respectively. (d2) Confocal microscopy of laser-affected tissue (rat liver dissection). (d3) Z-stacking of a region of interest (ROI) containing laser-affected tissue corresponding to 50% and 60% LPP. (e1) Raw images acquired from stain-free imaging of formalin-fixed rat tissues and *ex ovo* chorioallantoic membrane using second-harmonic generation. (e2) Magnified view of the region of interest within the raw image. The red arrows indicate renal collecting tubules and vasculatures containing erythrocytes in rat kidney cortex and *ex ovo* chorioallantoic membrane, respectively.

Two-photon microscopy is frequently applied for three-dimensional visualization of biological tissue [29]. Here, we test the two-photon microscopy to enable three-dimensional visualization of micro-agents by conducting Z-stacking experiments (Fig 3(a1) and 3(b1)). The beaded fiber and a scaled version of CeFlowBot (scale factor of 0.5) are imaged with 50× and 10× microscope objectives and spacing steps of 1 μm and 5 μm, respectively. The Z-stacking images, which are obtained using 20% LPP, are analyzed through a 3D slicer software for three-dimensional reconstructions (Fig 3(a2) and 3(b2), respectively) [42]. Furthermore, scanning electron microscopy of both micro-agents is included to validate the three-dimensional reconstructions and provide further morphology details (Fig 3(a3) and 3(b3)). Two-photon microscopy is experimentally validated for two-/three-dimensional visualization of fluorescent micro-agents. Yet, experiments involving biological tissue require further analysis regarding laser-tissue interaction to reduce the risk of side effects under *in vitro* and *in vivo* conditions.

### Laser-tissue interaction

Visualization of micro-agents below biological tissue using a near-infrared femtosecond pulsed laser holds the risk of tissue damage by laser ablation and cellular necrosis under live imaging conditions [43]. Here, we present a qualitative study of laser-tissue interaction to identify the minimum average power to generate tissue damage by laser ablation. Current studies on two-photon microscopy suggest average laser powers in the order of 100 mW (at 80 MHz and pulse width from 1 fs to 10 ns) to avoid optical breakdown [44]. Here, we analyze the tissue damage by laser ablation generated by our femtosecond pulsed laser (15 MHz repetition rate and 230 fs pulse width). Fig 3(c) shows the average power of the femtosecond pulsed laser by varying the laser power percentage (LPP).

The tissue damage by laser ablation is a photo-thermal effect that depends on the optical properties of tissue (e.g., absorption and reduced scattering coefficient) [45]. We use a formalin-fixed tissue (rat liver dissection) for laser ablation experiments. Three different LPPs (LPP = 40%, 50%, 60%) are employed with a spacing of 50 μm between lines to analyze the tissue damage by laser ablation (Fig 3(d1)). The laser-affected tissue is imaged using a confocal microscope for analysis. A visual inspection shows that 40% of LPP does not generate tissue damage by laser ablation compared to 50% and 60% LPPs (Fig 3(d2)). Such a result suggests that experiments along with biological tissue must be carried out using an LPP ≤ 40% (85 mW average power). Fig 3(d3) shows the Z-stacking (10 μm depth) within a region of interest containing laser-affected tissue corresponding to 50% and 60% LPP. It is worth noting that there is an increase in the absorption and reduced scattering coefficients of tissue after formalin fixation [46]. Thus, further experiments on laser-tissue interaction are required to validate our microscope in clinically-relevant scenarios.

In order to test the microscope under *ex vivo* and *ex ovo* conditions, four biological tissues are visualized using a stain-free approach and 20% LPP (35 mW average power) (Fig 3(e)). Stain-free imaging of biological tissue includes autofluorescence and second-harmonic generation according to excitation/emission spectra of proteins or cells in biological tissue [47]. Fig 3(d) shows the micrographs of fixed tissues and *ex ovo* chorioallantoic membrane containing erythrocytes within the vasculatures. Two-photon microscopy of biological tissue is tested using the maximum gain of the multiplier and gradually decreasing the value of LPP from 40% to 5%. Our results show that using an LPP ≤ 10% (6 mW average power) does not trigger the fluorescence intensity required for image formation.

### Visualization of micro-agents below fixed tissue

**Fig 4:**
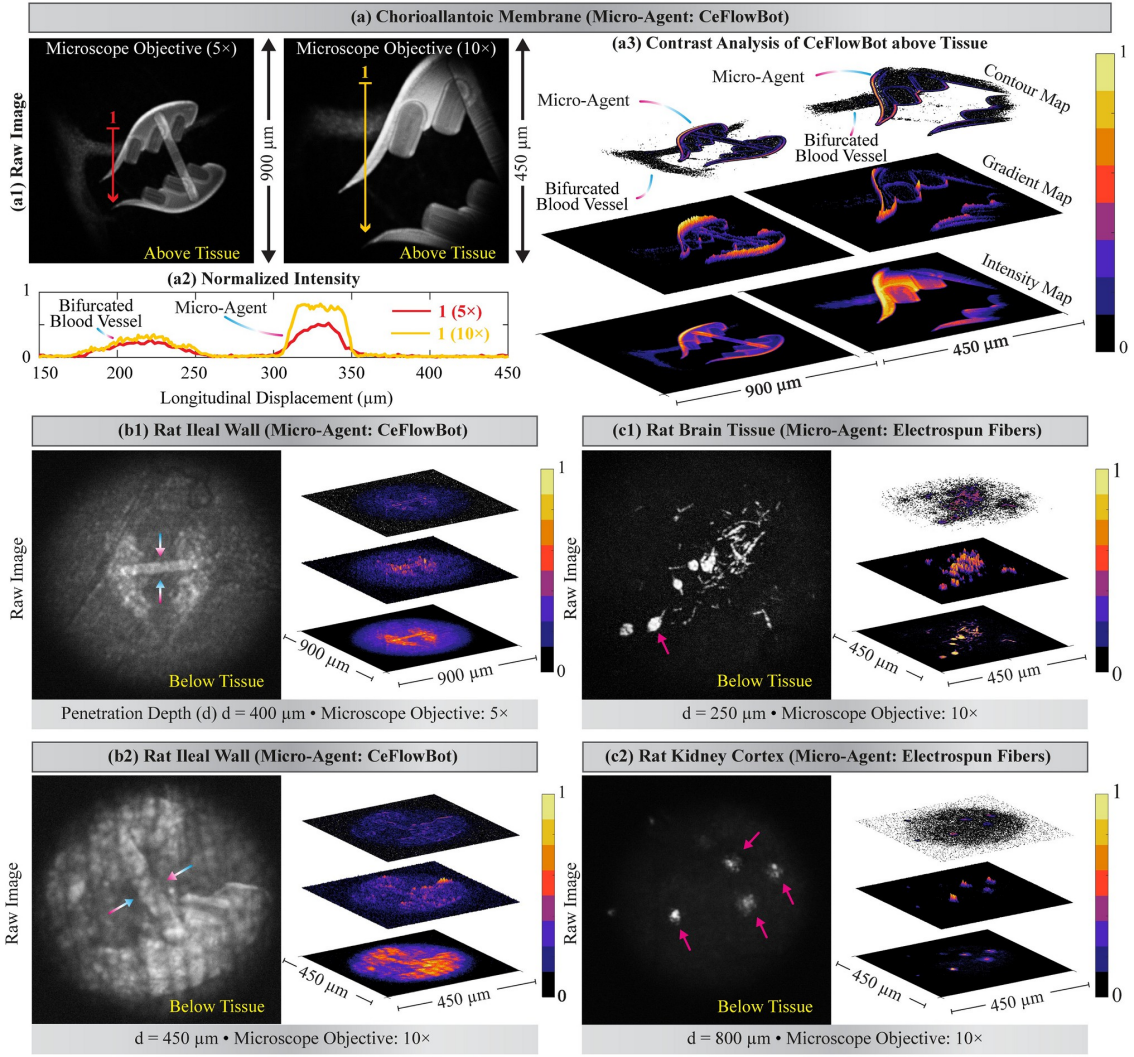
Contrast analysis of the two-photon microscopy images containing micro-agents and biological tissue. (a) Two-photon microscopy of CeFlowBot and a bifurcated vessel containing erythrocytes from *ex ovo* chorioallantoic membrane. (a1) Raw images acquired with 5× and 10× microscope objectives. (a2) Normalized fluorescence intensity of micro-agent and a bifurcated blood vessel along a longitudinal line. (a3) Contrast analysis to identify CeFlowBot and a bifurcated blood vessel. Two-photon microscopy and contrast analysis of CeFlowBot below rat ileal wall (fixed) using 5× (c1) and 10× (c2) microscope objectives. Two-photon microscopy and contrast analysis of electrospun fibers below rat brain tissue (fixed) (b1) and rat kidney cortex (fixed) (b2). The colored arrows indicate structural morphologies that are visible below fixed tissue.

The spectrum analysis of fluorescent micro-agents and the qualitative study of laser-tissue interaction pave the way for the visualization of micro-agents along with biological tissue. The two-photon microscope used in this study has a single channel for fluorescence acquisition. Thus, the visualization of micro-agent and tissue relies upon the difference between the intensity of fluorescence signals. Contrast analysis provides information about the micro-agent and physical surroundings by extracting contour, gradient, and intensity maps from the acquired images (Fig 4). The CeFlowBot is initially imaged above of an *ex ovo* chorioallantoic membrane to illustrate our method (Fig 4(a)). Two microscope objectives (5× and 10×) are utilized to modify the resolution and analyze the influence of the depth of field on the generation of fluorescence emission light (Fig 4(a1)). We observe that using the 10× microscope objective increases the image resolution allowing for detailed visualization of CeFlowBot morphology. Fig 4(a2) shows the normalized fluorescence intensity of CeFlowBot and *ex ovo* chorioallantoic membrane along a longitudinal line. The depth of field of the 10× microscope objective (3.5 μm) compared to the 5× microscope objective (14 μm) allows for more localized excitation of CeFlowBot, resulting in increased fluorescence emission light generation (Fig 4(a2)). Contrast analysis allows for the identification of CeFlowBot and bifurcated blood vessel (containing erythrocytes) from the *ex ovo* chorioallantoic membrane (Fig 4(a3)).

Experiments to visualize CeFlowBot below rat ileal wall (fixed) are carried with a maximum laser power percentage (LPP) of 40% to reduce the risk of tissue damage and demonstrate the potential of two-photon microscopy towards *ex vivo* and *in vivo* applications. Fig 4(b1) and 4(b2) show the visualization of CeFlowBot below the rat ileal wall using 5× and 10× microscope objectives. Our results reveal the structural morphology of CeFlowBot and provide visualization of the physical surroundings (e.g., muscularis propria) with penetration depths over 400 μm. For validating two-photon microscopy of both types of micro-agents, electrospun fibers are also imaged below formalin-fixed tissues with different reduced scattering coefficients (rat brain tissue and kidney cortex) (Fig 4(c1) and 4(c2)). The rat brain tissue has a reduced scattering coefficient much higher than other tissue samples throughout spectra (450–750 nm) [48]. Thus, the fluorescence emission light tends to scatter along the tissue sample, reducing the output image quality. The sample for visualization of electrospun fibers below rat brain tissue is prepared for imaging with a maximum penetration depth of 250 μm. Fig 4(c1) shows a detailed visualization of electrospun fibers (i.e., beaded fibers and rod-like particles) morphology below rat brain tissue. For imaging experiments of electrospun fibers along with rat kidney cortex, the samples are prepared with a maximum penetration depth of 800 μm. Fig 4(c2) shows two-photon microscopy and contrast analysis of electrospun fibers below the rat kidney cortex. Our results show that the optical scattering increases due to the penetration depth, yet the fundamental morphology of electrospun fibers is visualized via two-photon microscopy.

The images acquired from electrospun fibers below fixed tissue do not reveal further information about the physical surroundings since the fluorescence intensity of electrospun fibers is greater than the autofluorescence and second-harmonic generation in biological tissue. An appealing approach to overcome the challenges of using a single photomultiplier tube is to incorporate additional dichroic mirrors along with photomultiplier tubes for color-coded visualization [29]. Although our experimental validation platform has a single photomultiplier channel, our results uphold the visualization of micro-agents along with biological tissue via two-photon microscopy. Furthermore, we show that the micro-agents can still be detected using two-photon microscopy, displaying fundamental morphology despite changes in penetration depth and optical properties of fixed tissue.

### Continuous visualization of magnetic electrospun fibers

**Fig 5:**
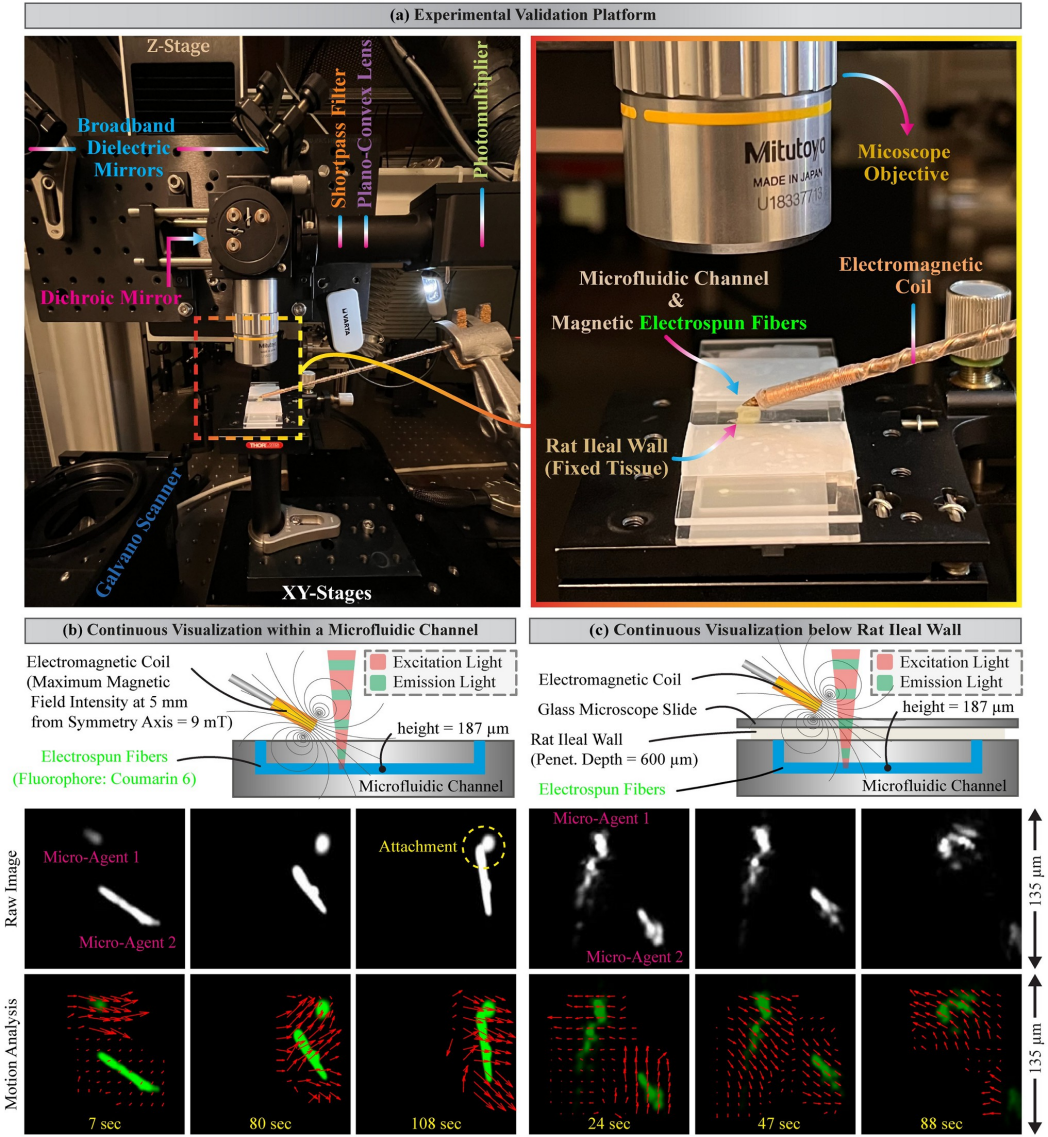
Actuation, continuous visualization, and motion analysis of magnetic electrospun fibers. (a) Experimental validation platform for two-photon image acquisition of micro-agents below biological tissue. (b) Visualization of magnetic electrospun fibers within a microfluidic channel using 10% laser power percentage (LPP) (6 mW average power). (c) Visualization of magnetic electrospun fibers below fixed tissue (rat ileal wall) using 40% LPP (85 mW average power). The experiments are carried out with a 10× microscope objective, a scanning speed of 50 mm/s, and a sampling rate of 10 MS/s. The vector field colored in red represents the motion analysis. The continuous image acquisition experiments are shown in S1 Video.

Static image acquisition of micro-agents below formalin-fixed tissue is the first step to bridging the gap between two-photon microscopy and microrobotics. However, the application domains of microrobotics require continuous imaging tools to localize micro-agents and verify their functionality. Here, we use two-photon microscopy for image acquisition of magnetic electrospun fibers at one frame per second in the field of view of 135 × 135 μm^2^ (Fig 5). A solution containing magnetic electrospun fibers is perfused into a microfluidic channel with a height of 187 μm made from polydimethylsiloxane (PDMS) [12]. Thereon, an electromagnetic coil is utilized for actuating the magnetic fibers (Fig 5(a)). In order to elucidate continuous imaging of micro-agents, we devise two experiments for motion analysis using Lucas-Kanade optical flow as vector fields [36, 37]. The first experiment shows two magnetic electrospun fibers (micro-agent 1 and 2) within a microfluidic channel (Fig 5(b)). The micro-agents are oriented and pulled according to the magnetic field and gradients. During the experiment, we show the attachment of both micro-agents due to electrostatic interaction. Electrostatic interactions are caused by residual charges embedded within the surface or volume of the electrospun fibers [49]. The micro-agents are detached once the magnetic field turns from a negative to a positive value, demonstrating the visualization of micro-agent functionalities via two-photon microscopy.

The second experiment is carried out by placing fixed tissue (rat ileal wall 600 μm thick) on top of the microfluidic channel and covering it with a glass microscope slide (Fig 5(c)). The results of this experiment uphold continuous imaging and actuation of two magnetic electrospun fibers below formalin-fixed tissue. In particular, fixed tissue is a dispersive media showing increments of reduced scattering coefficient compared to fresh tissue [46]. Therefore, visualization of electrospun fibers below fixed tissue requires a higher laser power percentage (LPP = 40%) for image formation compared to the previous experiment without tissue (LPP = 10%). Despite the increase in scattering, our results permit us to identify the morphology of electrospun fibers while providing continuous image acquisition for detection and visual tracking of micro-agents below biological tissue. The experiments are performed with an LPP ≤ 40% to reduce the risks of tissue damage by laser ablation.

One of the challenges observed in the experiments is the frame acquisition rate (1 fps), which imposes a limitation on the analysis of micro-agents with high dynamics. Previous *in vivo* studies have demonstrated that micro-agents can move at relatively low velocities (< 10 μm/s) under magnetic guidance [10, 50]. Drawing upon this comprehension, our two-photon microscope holds the potential for effective *in vivo* visualization. Furthermore, the incorporation of optical and optomechanical technologies, such as polygonal mirrors and resonant scanners coupled with optical fibers, can improve the scan rate of our two-photon microscope and enable real-time imaging [13, 51].

## Conclusion

This study introduces two-photon microscopy as an alternative visualization tool in microrobotics to overcome the penetration depth limitations of optical microscopy techniques frequently used in the application domains of microrobotics (i.e., widefield, brightfield, and confocal microscopy). This study demonstrates the use of two-photon microscopy for imaging micro-agents below formalin-fixed tissue. We present a qualitative study of laser-tissue interaction to provide information about the minimum average power for tissue damage by laser ablation. Our results display two-photon image acquisition of micro-agents with a spatial resolution of 1 μm and up to 800 μm penetration depth. Besides, we validate continuous imaging of micro-agents with a penetration depth of 600 μm to showcase the potential use of two-photon microscopy for visual tracking the micro-agents below biological tissue. Two-photon microscopy can enable visualization of micro-agents under *in vivo* and *in vitro* conditions by overcoming the limitations of existing optical imaging techniques for microrobotics.

## Supporting information

S1 Video Continuous visualization of magnetic electrospun fibers. Supplementary video showing the experiments on continuous visualization of magnetic electrospun fibers within a microfluidic channel and below fixed tissue (rat ileal wall).
(ZIP)
